# Mechano-induced persistent room-temperature phosphorescence from purely organic molecules†
†Electronic supplementary information (ESI) available: Synthetic procedures, experimental details and supplemental figures. CCDC 1581141. For ESI and crystallographic data in CIF or other electronic format see DOI: 10.1039/c8sc00429c


**DOI:** 10.1039/c8sc00429c

**Published:** 2018-03-19

**Authors:** Yingxiao Mu, Zhiyong Yang, Junru Chen, Zhan Yang, Wenlang Li, Xianbao Tan, Zhu Mao, Tao Yu, Juan Zhao, Shizhao Zheng, Siwei Liu, Yi Zhang, Zhenguo Chi, Jiarui Xu, Matthew. P. Aldred

**Affiliations:** a PCFM Lab , GDHPPC Lab , Guangdong Engineering Technology Research Center for High-performance Organic and Polymer Photoelectric Functional Films , State Key Laboratory of OEMT , School of Chemistry , Sun Yat-sen University , Guangzhou , 510275 , China . Email: ceszy@mail.sysu.edu.cn; b Department of Chemistry , Durham University , DH1 3LE , UK

## Abstract

The persistent room-temperature phosphorescence (RTP) of purely organic materials in the solid state has recently attracted much research interest and found promising, rapid and visual applications by the naked eye, after the removal of the excitation source. However, almost all reported organic persistent RTP processes are induced by using a UV-light irradiation source. In this report, persistent RTP with an emission lifetime as long as 0.15 s which can be induced not only by photoirradiation but also by mechanical action is presented, which merges mechano-induced luminescence and persistent RTP together. It is found that such persistent RTP is produced through intermolecular electronic coupling (IEC) of units with different excited state configurations. Interestingly, there are two different crystals with and without mechano-induced persistent RTP which can be grown from this organic material, as such a new type of mechano-luminescence (ML) is strongly dependent on their intermolecular interactions. Furthermore, the intensity of such mechano-induced persistent RTP can be increased by lowing the temperature as well, similar to that of photo-induced persistent RTP. Notably, these two crystals exhibit a ML enhancement and ML “turn on”, respectively, with decreasing temperature. Therefore, such mechano-induced persistent RTP provides an example of new types of organic luminescent materials, which is a missing jigsaw piece of organic luminescence and important for both fundamental research and practical applications of both persistent RTP and ML of organic materials.

## Abstract 2

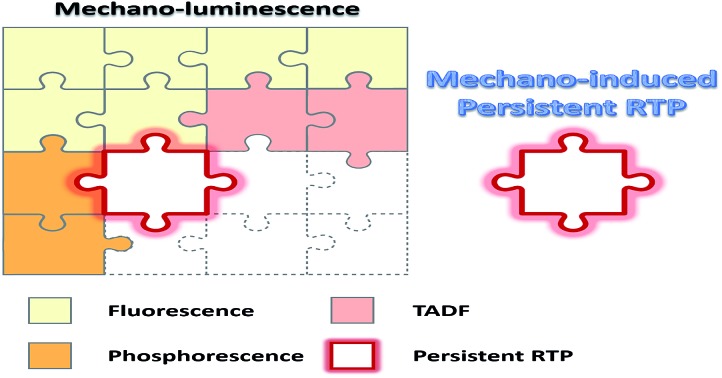
Interesting purely organic persistent room-temperature phosphorescence induced by not only photoirradiation but also mechanical action has been presented.

## Introduction

Organic luminescent materials, whose luminescence characteristics can be readily tuned not only by the design of the molecular structure but also by the control of intermolecular interactions in the solid-state, have attracted considerable interest and offered numerous advanced applications in organic lasers, organic light-emitting diodes (OLEDs), sensors and bioimaging.^1^ The emission process involved in a luminescent organic molecule is actually a transition between different excited states of the excitons. Therefore, the tuning and control of the exciton properties of organic materials, including their energy level, concentration, configuration, lifetime, and energy transfer, is an intrinsic determinant for their luminescence characteristics.^2^ In general there are two excited state configurations in organic molecules, namely singlet and triplet excitons, which exhibit different spin quantum numbers. The ground state configuration of organic molecules is generally a singlet state. Luminescence from triplet excitons possesses longer lifetimes because the electronic transition between triplet and singlet states (intersystem crossing, ISC) is spin-forbidden. Due to the longer lifetimes triplet excitons are easily quenched by environmental influences, such as heat and oxygen in air, of which the latter exhibits a triplet ground state at a very low energy level that can easily quench the other triplet excitons *via* Dexter energy transfer. Therefore, it is difficult to achieve bright emission from long-lived triplet excitons at room-temperature in air, such as organic RTP, due to such a conflict between strong emission and long lifetime for organic materials.^3^ Interestingly, there have been some recent reports demonstrating the persistent RTP of purely organic materials (metal-free) with extended emission lifetimes as long as hundreds of milliseconds in the crystalline state by the promotion of ISC from the lowest singlet excited state (S_1_) to the lowest triplet excited state (T_1_).^4^ Although the exact mechanism about such a long radiative process of triplet excitons is still unclear, the highly increased studies^5^–^9^ on this interesting organic luminescence phenomenon have revealed that intermolecular interactions in crystals have an important effect on such bright and long-lived RTP, such as electronic couplings in different molecular stacking arrangements to promote the ISC transition (H-aggregates,^5^ n–π^6^ or π–π^7^ stacking) and weak interactions^8^ (CH–π) to rigidify the molecules in the crystalline state. These new types of organic luminescent materials provide deep insights regarding the tuning and control of excitons, which is one of the most important issues of the fundamental investigations of organic luminescent materials. Furthermore, due to the long-lived emission, researchers have also found promising visual applications, such as multiple stimuli (force, temperature, and solvent vapor) sensors,8a,9a data encryption,5a,6a and bioimaging,5b,6b,9b in which persistent RTP can be observed by the naked eye after the removal of the excitation source. Nevertheless, all the reported organic persistent RTP materials are photo-induced emissive and generally excited by using an extra UV-light irradiation source, which can be phototoxic for bio-applications and difficult for monitoring *in situ*. Compared to photoluminescence (PL), mechano-luminescence (ML, also known as triboluminescence), which is induced by mechanical stimuli, such as grinding, scratching, squeezing or shaking, has attracted extensive attention owing to its advantageous environmentally friendly characteristics and good compatibility with *in situ* detection.^10^ However, the number of purely organic materials with ML properties is limited and they generally exhibit mechano-fluorescence. It is rare for organic ML materials to exhibit mechano-phosphorescence properties,^11^ let alone mechano-induced persistent RTP. Therefore, if the emission from an organic material can be triggered by mechanical stimuli, persistent RTP will have more diverse applications, such as bio-applications. In addition, a practical sensor system with the advantages of being able to be visualized by the naked eye, *in situ* and in real-time, could be realized for different advanced applications, as well as providing insight into the exciton properties of organic materials.

**Fig. 1:**
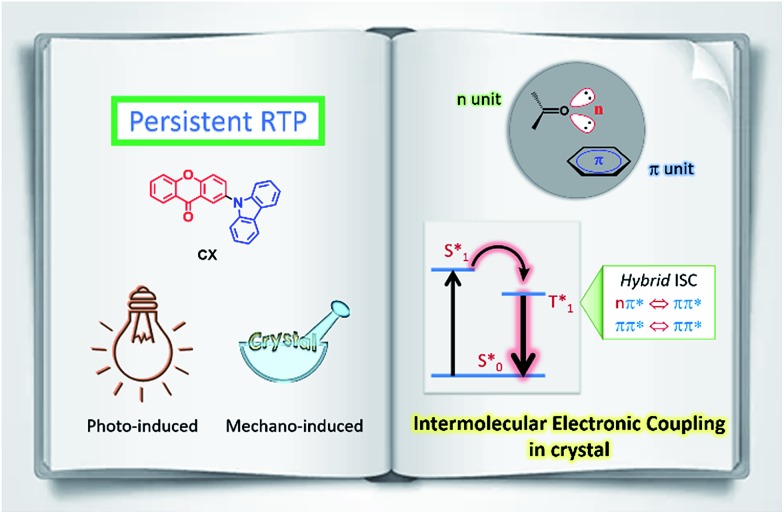
Schematic diagram of the intermolecular electronic coupling in the crystalline state for persistent RTP: (left) chemical structure of the compound CX, (above right) intermolecular n- and π-units stacked in the crystal, and (below right) hybrid intersystem crossing formation. 
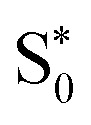
 = the ground state, 
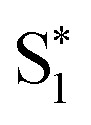
 = the lowest singlet excited state, and 
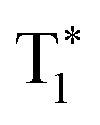
 = the lowest triplet excited state. All these states refer to the proposed intermolecular electronically coupled states.

In this communication, an example of a purely organic molecule (CX) with mechano-induced persistent RTP is reported and studied in detail. This organic material exhibits persistent RTP from triplet excitons with a lifetime as long as 0.15 s. As shown in Fig. 1, this long-lived RTP originates from the intermolecular electronic coupling (IEC) formation of n and π units in the crystalline state, which possess different electronic configurations ((n, π*) and (π, π*)) of their T_1_ excited states^12^ and, therefore, produce a hybrid ISC for persistent RTP. It is noted that such persistent RTP can be induced by either UV-light irradiation or mechanical action (Fig. 1). In other words, CX exhibits mechano-induced persistent RTP, which is a new type of luminescence and the study of such a process is important in terms of both fundamental research and practical applications.

## Results and discussion

As shown in our previous reports, intermolecular n–π stacking in the crystalline state is one of the efficient approaches of IEC formation for persistent RTP, in which n- and π-units are generally donor (D) and acceptor (A) groups.^6^ The carbazolyl (Cz) unit is an excellent π-unit and has previously been used successfully to construct D–A type molecules with different n groups for persistent RTP materials, because its T_1_ state with the (π, π*) configuration facilitates slow radiative decay of T_1_ excitons.3*a* The xanthone (XTO) group exhibits a high ISC rate for promoting triplet excitons, making it a suitable n-unit (T_1_ state with the (n, π*) configuration) for persistent RTP molecular design. It is well known that a twisted D–A molecular structure can efficiently reduce the energy gap (Δ*E*_ST_) between the S_1_ and the T_1_ states, which results from the reduction of the spatial overlap between the highest occupied molecular orbital (HOMO) and the lowest unoccupied molecular orbital (LUMO). Additionally, it can increase the ISC rate for producing populated triplet excitons in molecules with small Δ*E*_ST_, which is widely utilized to design efficient thermally activated delayed fluorescence (TADF) materials. CX has a simple twisted D–A molecular structure, consisting of a π-unit (Cz) connected with an n-unit (XTO), and is readily synthesized by aromatic nucleophilic substitution between 2-fluoro-9*H*-xanthen-9-one and carbazole in good yield (66%). The chemical structure and purity were characterized by ^1^H and ^13^C nuclear magnetic resonance (NMR), high resolution mass spectrometry (HRMS), high performance liquid chromatography (HPLC) and elemental analysis (Scheme S1 and Fig. S1–7†).

**Fig. 2:**
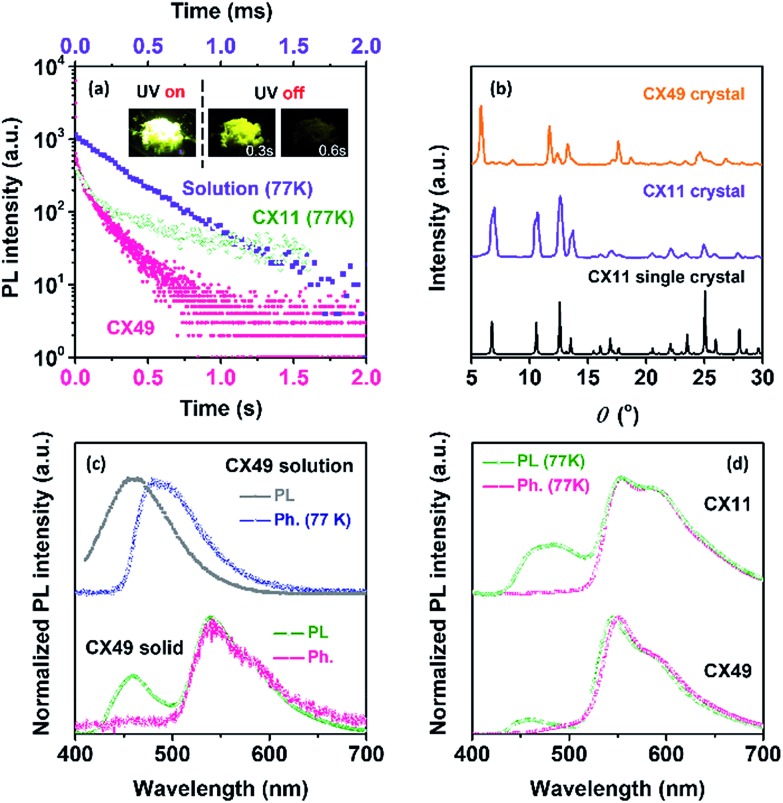
(a) Time-resolved emission decay curves of CX under different conditions: CX49 crystal (545 nm), CX11 crystal (545 nm, 77 K) and in toluene solution (485 nm, 10^–5^ M, 77 K, top coordinate). Inset photos are the CX49 crystal sample before (on) and after (off) the UV-light exposure removal. (b) Comparison of the powder X-ray diffraction spectra of CX49 and CX11 crystals, and the simulated spectrum from the CX11 single crystal. (c) Steady-state photoluminescence (PL) spectra and phosphorescence spectra (Ph, delayed 8 ms) of CX in toluene solution (10^–5^ M) and the crystalline (CX49) state. The spectra were recorded at room-temperature unless otherwise stated. (d) Steady-state PL spectra and Ph spectra (delayed 8 ms) of CX11 and CX49 crystals at 77 K.

As a D–A type molecule, CX exhibits broad charge transfer (CT) absorbance in the range of 350 to 400 nm and shows typical broad CT emission spectra in dilute solution, in which a bathochromic shift is observed when the solvent polarity is increased (Fig. S8a†). Density functional theory (DFT) calculation results in a vacuum also indicate that intramolecular CT exists in the CX molecule (Fig. S9a†). The dihedral angle between the Cz and XTO units is 55.3° from the optimized conformation, revealing a twisted molecular structure. As a result, its electrons are mainly spread in Cz and XTO groups for its HOMO and LUMO orbits, respectively, showing an intramolecular charge separation between its ground and excited states. In addition, the Δ*E*_ST_ of CX is found to be 0.21 eV by DFT calculation (Fig. S9b†), resulting in a small spatial overlap between its HOMO and LUMO. Actually, the Δ*E*_ST_ was measured to be 0.23 eV from the onsets of its fluorescence and phosphorescence spectra at 77 K, which is suitable for promoting ISC and thus RTP emission (Fig. S8b†). Furthermore, the n- and the π-units possess different instantaneous polarities, due to the intramolecular CT characteristics of CX. Therefore, the n- and the π-units of different CX molecules in their crystalline state should stack together (n–π stacking), forming IEC for the promotion of persistent RTP. When recrystallized from a mixed solvent system of dichloromethane (DCM) and *n*-hexane (4/9, v/v), an obvious yellow RTP emission (inset photos in Fig. 2a) can be observed from the CX crystals (CX49) after turning off the excitation UV-light source (365 nm), which by further analysis is confirmed to be persistent RTP.

As shown in Fig. 2c, the CX49 crystal shows both fluorescence and phosphorescence peaks in its steady-state spectra at room temperature with an overall quantum yield (QY) of 40%, compared to only fluorescence observed in dilute toluene solution. For the CX49 crystal, the fluorescence peak is located at 460 nm and exhibits a typical short lifetime of 7.2 ns (Fig. S8c and Table S2†), which is attributed to a fast-radiative decay process of the S_1_ excitons. However, the asymmetric peak of the CX49 crystal located at 545 nm possesses a long emission lifetime of 0.15 s, revealing a persistent RTP emission (Fig. 2a and Table S1†). The type of this asymmetric peak was further confirmed by the enormous increase of its intensity (∼25 fold, from ∼200 to ∼5000) as the temperature lowered from 300 to 77 K (Fig. 4a and Table S1†). Similar to other organic long-lived RTP materials,^4^ the persistent RTP of CX49 should come from IEC formation in its crystalline state, which is different compared to the phosphorescence (0.3 ms) of CX49 molecules in dilute toluene solution at 77 K, in terms of the emission peak wavelength or the phosphorescence lifetime (Fig. 2a and c, Table S2†). Although the single crystal of CX49 could not be obtained, another single crystal (CX11, QY = 31%) of the compound CX was obtained from a solvent mixture of DCM and *n*-hexane (1 : 1, v/v) by slow solvent evaporation, which exhibits, from powder X-ray diffraction (PXRD) studies, a different X-ray diffractogram compared to the CX49 crystal (Fig. 2b). Interestingly, the CX11 crystal exhibits no persistent RTP but only long-lived phosphorescence at low temperature (0.20 s, 77 K, Fig. 2a and Table S3†). Additionally, both the steady-state and the phosphorescence (delayed) spectra of the CX11 crystal are similar to those of the CX49 crystal at 77 K, indicating that they are alike in the triplet exciton process at low temperature, as shown in Fig. 2d. Interestingly, their persistent phosphorescence properties with regard to both the lifetime and the temperature sensitivity are different. The phosphorescence of the CX11 crystal exhibits a shorter lifetime and its intensity decreases much faster as the temperature increases than those of the CX49 crystal (Tables S1 and S3†). These results reveal that intermolecular interactions in the crystalline state have a significant effect on the long-lived phosphorescence or persistent RTP.

**Fig. 3:**
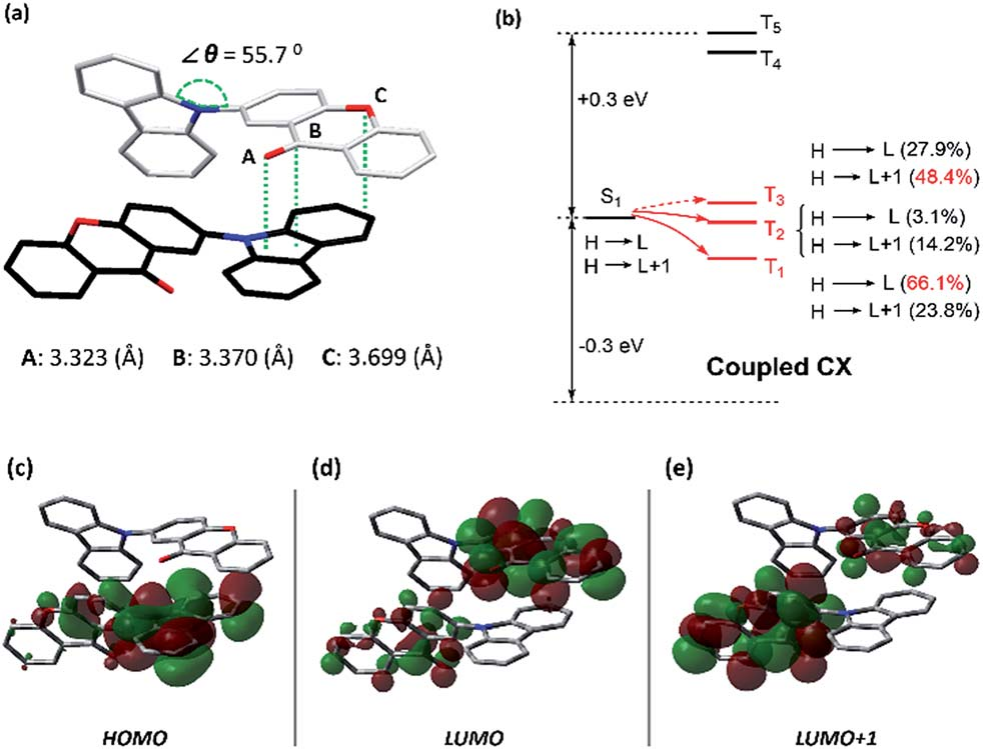
(a) Intermolecular interactions of the XTO and Cz groups in two neighboring CX molecules in the CX11 single crystal. The distances from the atoms (A, B and C) in the XTO group to the electronically coupled Cz plane are indicated by green dashed lines: (A) 3.323 Å, (B) 3.370 Å and (C) 3.699 Å. The twist angle between the XTO and Cz groups of the CX molecule is indicated as well. (b) Schematic representations of the TD-DFT calculated energy levels, main orbital configurations and possible ISC channels of coupled CX in the CX11 single crystal in the singlet (S_1_) and triplet (T_*n*_) states. The notations H and L refer to the HOMO and LUMO, respectively. The plain arrows and dashed arrows refer to the major and minor ISC channels, respectively. (c–e) The Kohn–Sham frontier orbitals that are involved in the probable ISC channel obtained from the electronically coupled CX molecules in the CX11 crystal.

Further insight into the persistent phosphorescence of the CX crystals was obtained by carefully analyzing the crystallographic structure of the CX11 single crystal. As shown in Fig. 3a, CX molecules adopt a twisted molecular conformation with a twist angle of 55.7° between the XTO and Cz units, which is very close to that of the DFT calculated result (55.3°) from free CX molecules in a vacuum. This twisted conformation also reduces the conjugation between the HOMO and LUMO of the CX molecules and thus results in a small Δ*E*_ST_ for CX molecules in the crystalline state (Fig. S10a†). Such a small Δ*E*_ST_ will increase the ISC rate to produce T_1_ excitons for facilitating phosphorescence. Additionally, the XTO and Cz units, *i.e.*, the n- and π-units in CX molecules, are stacking close to each other, which is beneficial for IEC formation in the crystalline state. As shown in Fig. 3a, the carbonyl group in the XTO unit adopts a roughly parallel stacking to the Cz group in a neighboring CX molecule with a short distance of 3.323 Å (distance A) for the oxygen atom and 3.370 Å (distance B) for the carbon atom. Another oxygen atom in the XTO group also stacks close to the Cz plane with a short distance of 3.699 Å (distance C). Moreover, lots of weak interactions (CH–π) can be found to rigidify CX molecules in its crystal (Fig. S11†). The results of time-dependent DFT (TD-DFT) calculations based on the isolated CX molecule and the coupled molecules of its single crystals also reveal the IEC formation between such n- and π-units in the crystal. For isolated CX molecules in their crystalline state, the ISC transition channel from S_1_ to T_1_ with a moderate Δ*E*_ST_ of 0.21 eV is calculated to be the only possible channel (Fig. S10b†). This result is almost the same as the TD-DFT calculated one of the free isolated CX molecule in a vacuum, because they exhibit similar molecular conformations (Fig. S9b†). In contrast, three possible ISC channels, from S_1_ to T_1_, T_2_ and T_3_, respectively, were revealed by TD-DFT calculations based on the electronically coupled CX molecules in their crystalline state (Fig. 3b). Additionally, all these channels contain the components (HOMO → LUMO and HOMO → LUMO + 1) of intermolecular ISC transitions, as either LUMO or LUMO + 1 orbits are spread to both CX molecules in this coupled system. Especially, the most possible ISC channel (S_1_ to T_1_) exhibits a very small Δ*E*_ST_, only 0.065 eV, which is much smaller than that of the isolated molecule. Furthermore, the main component of this ISC channel is an intermolecular transition from the Cz unit to the XTO unit in the adjacent molecule. All these calculated results suggest that the coupled CX molecules in the crystalline state are helpful to improve ISC transition and thus T_1_ exciton population for persistent phosphorescence.

**Fig. 4:**
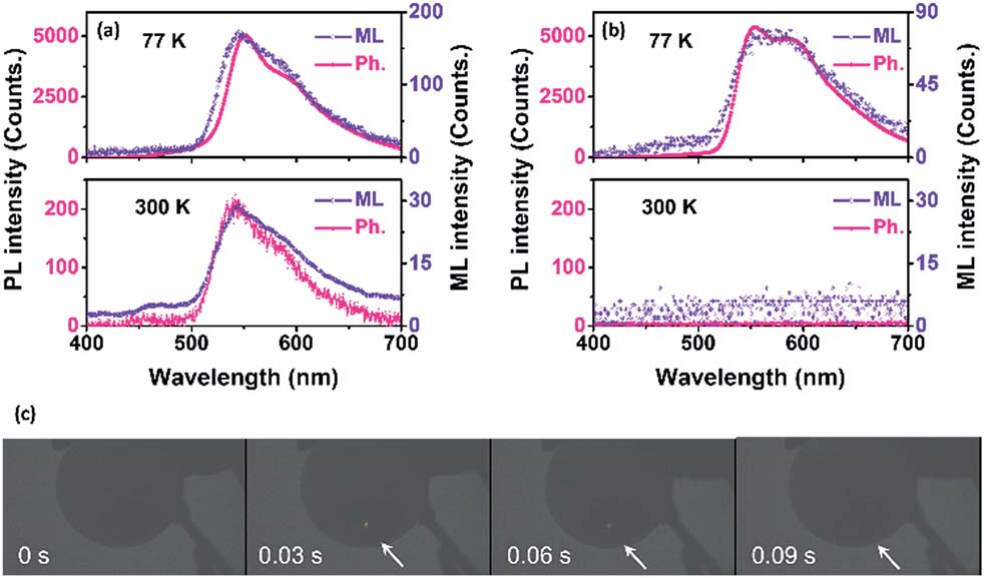
Comparison of the phosphorescence (Ph) and mechano-luminescence (ML) spectra of (a) CX49 and (b) CX11 crystals at 300 and 77 K. (c) ML photos of the CX49 crystal at 77 K, showing mechano-induced persistent phosphorescence.

Most interestingly, such persistent RTP can not only be photo-induced but also be mechanically induced, which is rare and different from the previous reports demonstrating persistent RTP in purely organic materials.^4^ An obvious yellow glow can be observed from CX49 crystals, when the sample is simply scratched at room temperature (Fig. S11 and Video S1†). As shown in Fig. 4, it is noted that such long-lived mechano-induced yellow luminescence becomes brighter and can still be observed after stopping the mechanical agitation (delayed ∼30 ms), when the scratching is carried out at 77 K (Fig. S13 and Video S2†). This is totally different from the typical organic materials with mechano-fluorescence (for example, *p*-P_4_A),11*c* in which the delayed fluorescent spot cannot be observed under the same conditions (Fig. S14 and Video S3†). Furthermore, the collected ML spectrum with an emission peak at 545 nm is similar to that of the photo-induced RTP in contrast to the steady-state PL spectra of CX49. In the ML process molecules are excited and emit light under mechanical action (pressure or shear force) and electric action (piezoelectricity or triboelectrification) when the crystals fracture.10a,11a It is common to observe differences between the ML and the relative PL spectra due to many reasons.11*a* Among these reasons, it seems that the self-absorption of the fluorescence band is an important reason for such differences in the CX49 crystal, which is indicated by the partial overlap between its UV-Vis absorption and PL spectra (Fig. S8d†). Especially, such ML in the CX49 crystals is a persistent RTP emission which can be observed by the naked eye. Therefore, except for fluorescence and phosphorescence reported in the previous literature,10a,^11^ persistent RTP can also be induced by mechanical stimuli. It is noted that this mechano-induced persistent RTP is actually a new type of intermolecular ML for purely organic materials, which originates from intermolecular interactions, as mentioned above. For such a new type of intermolecular ML, the spectrum of mechano-induced persistent phosphorescence exhibits good similitude to the relative photo-induced persistent phosphorescence, both at room-temperature and low temperature (Fig. 4a and b). Additionally, the intensity of the mechano-induced persistent phosphorescence also increases, the same as what occurs for the aforementioned photo-induced persistent phosphorescence with decreasing temperature. These results reveal that the produced persistent phosphorescence is similar, induced either by UV-light irradiation or by mechanically destroying the crystals. Intriguingly, the intermolecular interactions also have a significant effect on such mechano-induced persistent RTP. Another crystal of CX (CX11), which shows no photo-induced persistent RTP, exhibits no mechano-induced persistent RTP as well. However, its persistent ML will “turn on” at low temperature (Fig. 4b), while the persistent ML intensity of the CX49 crystal increases ∼6 fold when cooled down to 77 K (Fig. 4a). Therefore, two different temperature sensors, which are visualized by the naked eye, *in situ* and in real-time, could be expected when using these different mechano-induced RTP crystals.

## Conclusions

In summary, we have reported interesting persistent RTP from a purely organic material, which can be induced by not only photoirradiation but also mechanical action. This luminogen, namely CX, shows persistent RTP from triplet excitons with an emission lifetime as long as 0.15 s in its crystalline state at room temperature. It was demonstrated that this long-lived RTP originates from the IEC of the stacked n- and π-units in the crystalline state, which have different T_1_ state configurations with (n, π*) and (π, π*). As a result, such mechano-induced persistent RTP is also a new type of ML originating from intermolecular emission. Notably, this mechano-induced persistent RTP is strongly dependent on these intermolecular interactions, resulting in two different CX crystals with and without mechano-induced persistent RTP. Furthermore, both crystals exhibit an enhancement in the intensity of mechano-induced phosphorescence with decreasing temperature: one is a ML enhancement, while the other is ML “turn on”. As an emission merged with ML and persistent RTP, this type of mechano-induced persistent RTP could have applications in different monitoring systems with the advantages of being able to be visualized by the naked eye, *in situ* and in real-time. This study also provides an example of an organic compound with a new type of luminescence, which is a missing jigsaw piece of organic luminescence and important for both fundamental research and practical applications of organic materials.

## Conflicts of interest

There are no conflicts to declare.

## Supplementary Material

Supplementary information

Supplementary movie

Supplementary movie

Supplementary movie

Crystal structure data
