# Supplementary material for: Mechano-induced persistent room-temperature phosphorescence from purely organic molecules
Source: Chem Sci. 2018 Mar 19;9(15):3782–7. doi: 10.1039/c8sc00429c (PMC5939606; doi:10.1039/c8sc00429c)
Supplement: Supplementary file 1 [file SC-009-C8SC00429C-s001.pdf]

Electronic Supplementary Material (ESI) for Chemical Science.
This journal is © The Royal Society of Chemistry 2018

Mechano-induced persistent room-temperature phosphorescence from purely organic molecule

Yingxiao Mu,† Zhiyong Yang,† Junru Chen, Zhan Yang, Wenlang Li, Xianbao Tan, Zhu Mao, Tao Yu, Juan Zhao, Shizhao Zheng, Siwei Liu, Yi Zhang,* Zhenguo Chi, Jiarui Xu and Matthew. P. Aldred

Contents

1. **General Experimental Procedures.**
2. **Synthesis.**
3. **Structural Characterization Information.**
4. **Optical Properties.**
5. **Time-Dependent Density Functional Theory (TD-DFT) Calculations.**
6. **Molecular Stacking and Intermolecular Interactions in the Crystalline State.**
7. **Mechanoluminescence Photos.**
8. **References.**

1. General Experimental Procedures

Materials

2-chlorobenzoic acid, 4-fluorophenol, cuprous iodide, carbazole and *tert*-butoxide (*t*-BuOK) were purchased from Alfa Aesar and used as received. Ultra-pure water was used during the work-up procedures in the experiments. All other reagents and solvents (analytical grade) were purchased from Guangzhou Jincheng Company (China) and used without further purification. Crystals of CX49 and CX11 were grown from a dichloromethane (DCM)/*n*-hexane mixture by slowly solvent evaporation.

Characterization

Proton and carbon NMR (^1H NMR and ^{13}C NMR) spectra were obtained on a Fourier transform nuclear magnetic spectrometer (Bruker AVANCE 400) ($CDCl_3$, tetramethylsilane (δ = 0) as the internal standard). The mass spectra were measured using thermo-spectrometers (DSQ & MAT95XP-HRMS). The elemental analysis was conducted using an Elementar Vario EL analyzer. The high performance liquid chromatography (HPLC) was measured by a Thremo LCQ DECA XP liquid chromatography spectrometry. The UV-visible absorption spectrum was determined on a Hitachi U-3900 spectrophotometer. The photoluminescence (PL) spectra were collected with a spectrometer (FluoroLog-3) from HORIBA Instruments or a spectrometer system (Maya Pro2000) form Ocean Optics with a 310 nm Rhinospectrum RhinoLED as the excitation source. The persistent phosphorescent spectra were detected at a delayed time of 8 ms after the excitation source turn off. The measurements of mechanoluminescent spectra were carried out on a spectrometer of Acton SP2750 with a liquid-nitrogen-cooled CCD (SPEC-10, Princeton) as a power

detector. The mechanoluminescent photos were taken using a Canon EOS 700D camera (18000k pixel) in a REC mode, which frame number per second is 30. The first spot in the frame was set as the beginning of the ML for each scratching. Powder wide-angle X-ray diffraction (PXRD) measurements were performed on an X-ray diffractometer (Smartlab) using an X-ray source of Cu Kα (λ = 0.15406 nm) at 40 kV and 30 mA, at a scan rate of 4° (2θ) per min. The quantum chemistry calculations were performed at the B3LYP/6-31G (d) level of theory using the time-dependent density functional theory (TD-DFT) method with the Gaussian 09 software.

The single-crystal X-ray diffraction data were collected using a Gemini A Ultra system (Agilent Technologies) or a Smart 1000 CCD (Bruker), with Cu-Kα radiation (λ = 1.54178 Å). Both structures were solved using direct methods following the different Fourier syntheses. By using the SHELXTL program suite, all non-hydrogen atoms were anisotropically refined through least-squares on F^2 and assigned with the anisotropic thermal parameters. Meanwhile, the hydrogen atoms attached to carbon were placed in idealized positions and refined using a riding model. CCDC 1581141 contains the crystallographic data of crystal CX11 in this paper, respectively.

2. Synthesis

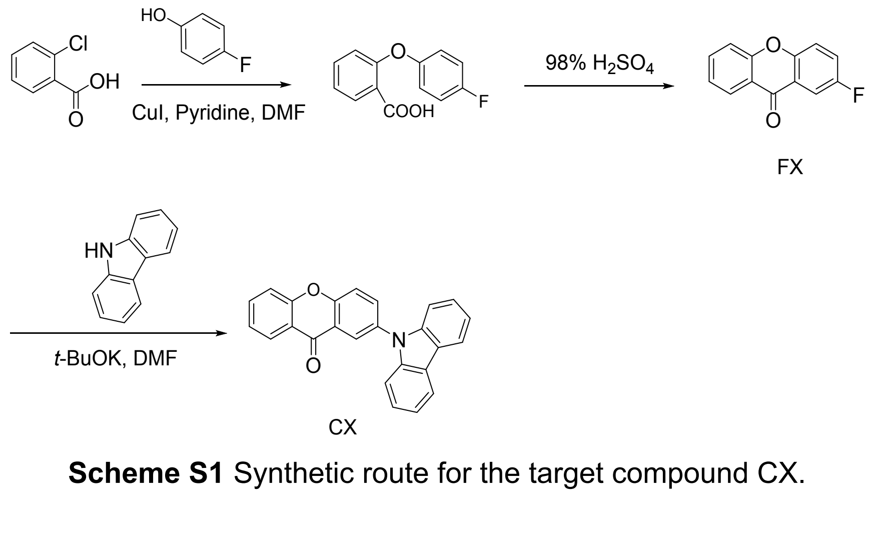

Scheme S1 Synthetic route for the target compound CX.

Synthesis of 2-fluoro-9H-xanthen-9-one (FX)

Cuprous iodide (0.18 g, 0.96 mmol) and pyridine (1.54 mL, 19.16 mmol) were added to a solution of 2-chlorobenzoic acid (3.00 g, 19.16 mmol), and 4-fluorophenol (4.30 g, 38.32 mmol) in *N*,*N*-dimethylformamide (DMF, 50 ml). The resulting solution was heated at 150 °C for 5h. The reaction mixture was cooled to room temperature and poured into a diluted HCl solution. The precipitate was filtered, and washed several times with water in order to remove any leftover acid. The crude product was collected without further purification.

The above product was dissolved in concentrated sulfuric acid (50 ml). The resulting suspension was stirred for 6 h at 100 °C. After cooling to room temperature, the mixture was poured slowly over ice. The precipitate was filtered, and washed several times with water in order to remove any leftover sulfuric acid. The collected solid was then re-dissolved in dichloromethane and washed with water (20 ml × 3). The combined water layers were extracted with additional dichloromethane. Then the combined organic extracts were dried over anhydrous sodium sulfate. After filtration and solvent evaporation under reduced pressure, a white solid was obtained in 78.1% yield (2.53 g). ^{1}H NMR (400 MHz, $CDCl_3$) δ (ppm): 8.37-8.26 (dd, 1H), 7.99-7.94 (dd, 1H), 7.77-7.70 (m, 1H), 7.53-7.36 (m, 4H). EI-MS, *m/z*: $[M]^+$ 214; calcd. for $C_{13}H_7FO_2$, 214.

Synthesis of 2-(9H-carbazol-9-yl)-9H-xanthen-9-one (CX)

To a solution of carbazole (0.94 g, 5.60 mmol) in dry DMF (50 ml), potassium *tert*-butoxide (1.57 g, 14.01 mmol) was added slowly. After the solution was stirred at room temperature under an argon atmosphere for 20 mins, FX (1.00 g, 4.67 mmol) was added. Then the reaction mixture was heated to 110 °C and stirred for an additional 6 h. After cooling to room temperature, the mixture was poured into 500 ml ice/water mixture, and the formed precipitate was filtered and washed with water (50 ml × 3) several times. The crude product was then

recrystallized from dichloromethane/acetone to produce a yellow solid in 66.4% yield (1.12 g). ^{1}H NMR (500 MHz, $CDCl_3$) δ (ppm): 8.58-8.51 (d, 1H), 8.39-8.34 (dd, 1H), 8.18-8.11 (d, 2H), 7.93-7.89 (dd, 1H), 7.80-7.76 (m, 1H), 7.75-7.72 (d, 1H), 7.59-7.56 (d, 1H), 7.45-7.38 (m, 5H), 7.32-7.28 (m, 2H). ^{13}C NMR (100 MHz, $CDCl_3$) δ (ppm): 176.62, 156.24, 154.86, 140.82, 135.23, 133.80, 133.70, 126.88, 126.19, 124.79, 124.39, 123.55, 122.97, 121.60, 120.44, 120.36, 120.01, 118.12, 109.50. EI-MS, m/z: [M]+ 361; calcd. for $C_{25}H_{15}NO_2$, 361. HRMS, m/z: [M]+ 361.1094; calcd. for $C_{25}H_{15}NO_2$, 361.1097. Anal. calcd. for $C_{25}H_{15}NO_2$: C 83.09, H 4.18, N 3.88; found: C 83.12, H 4.22, N 3.85. Two Crystals were grown from DCM/*n*-hexane mixture by solvent evaporation at room temperature: crystal CX49 (DCM/*n*-hexane = 4:9, v/v) and crystal CX11 (DCM/*n*-hexane = 1:1, v/v). And the crystal CX11 was suitable for X-ray analysis.

3. Structural Characterization information

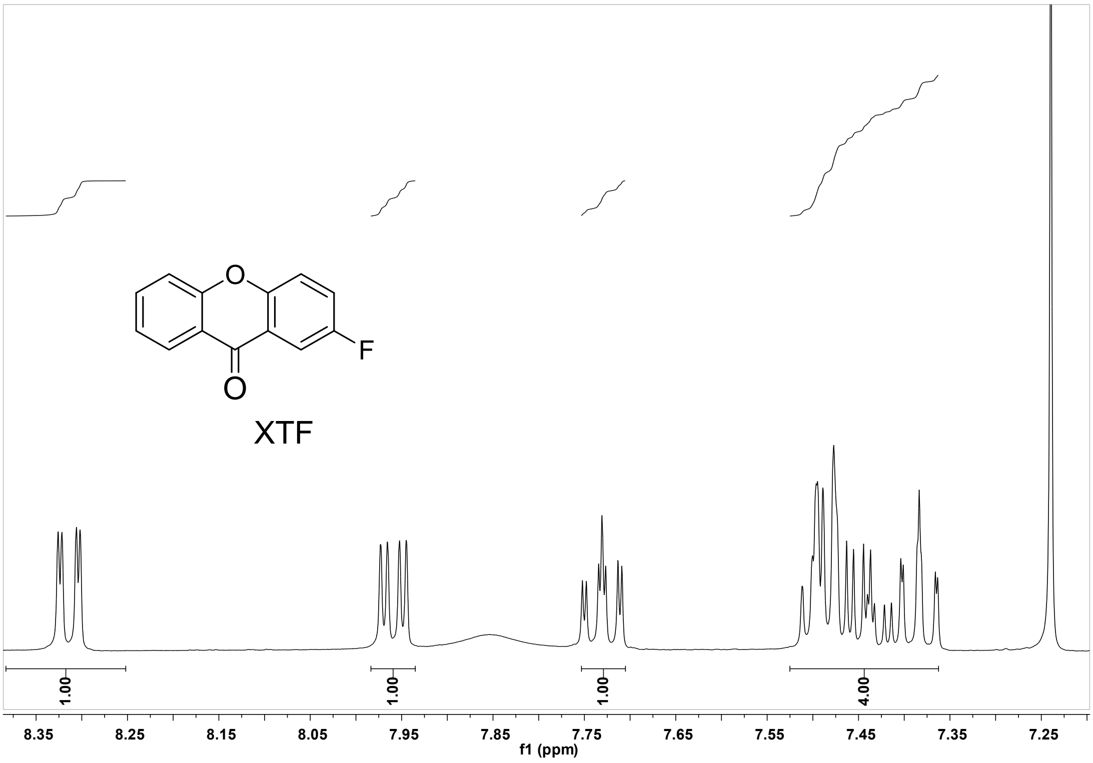

Fig. S1 ^{1}H NMR spectrum of compound FX.

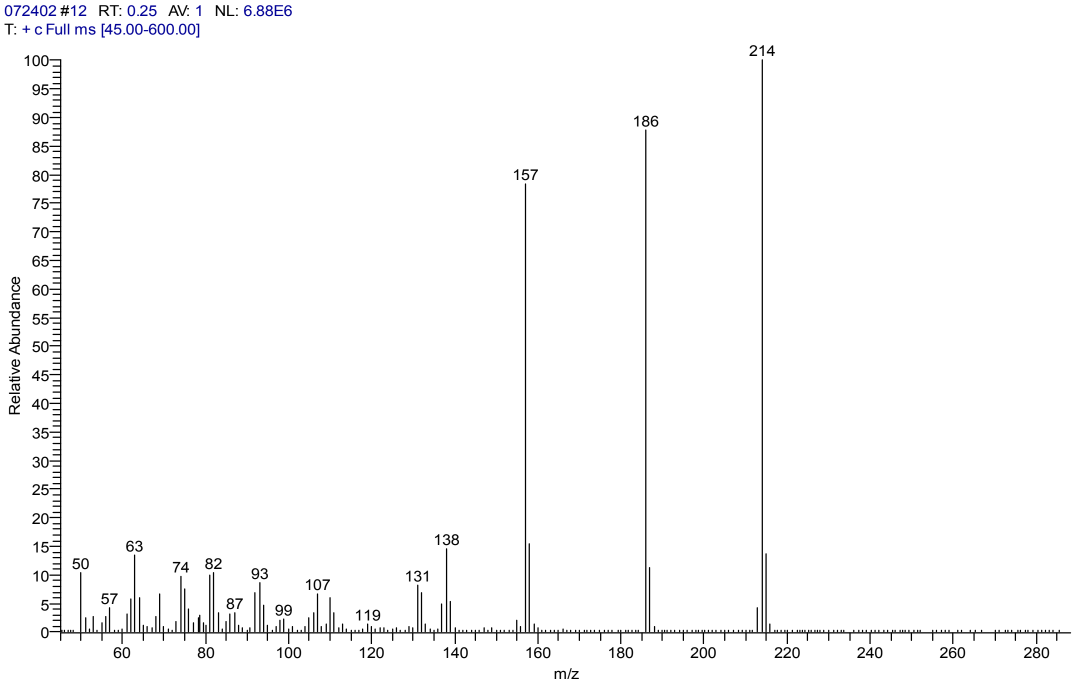

Fig. S2 EI-MS spectrum of compound FX.

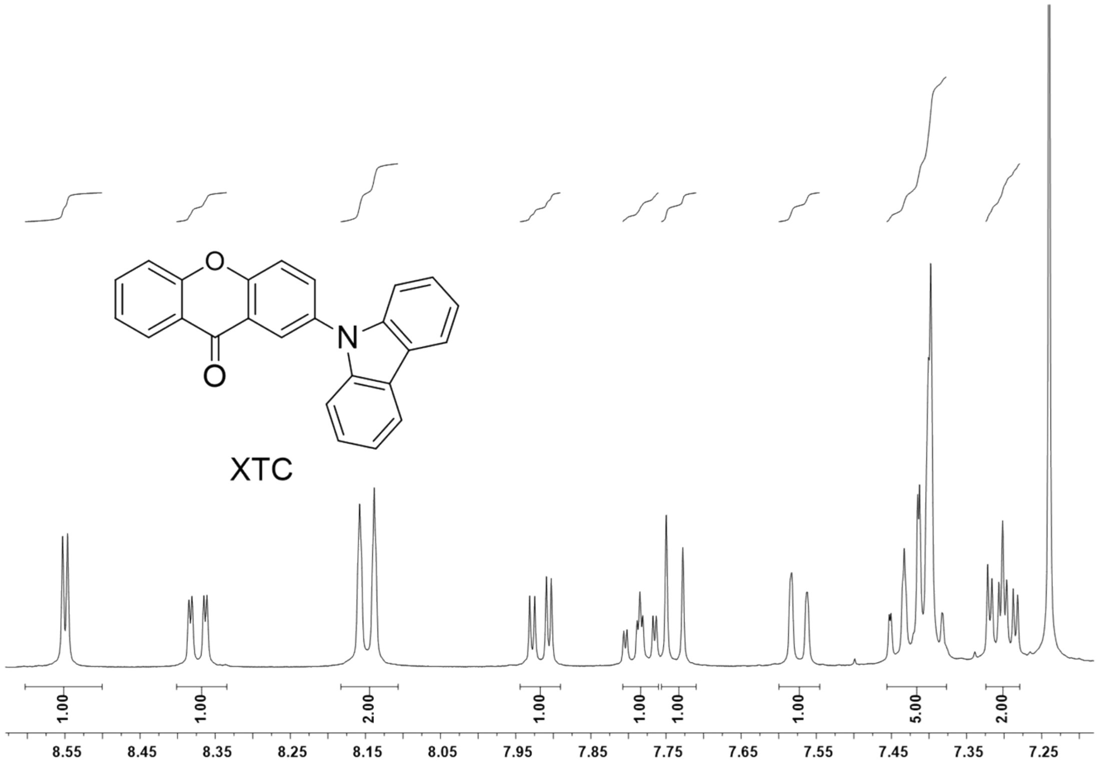

Fig. S3 ^{1}H NMR spectrum of compound CX.

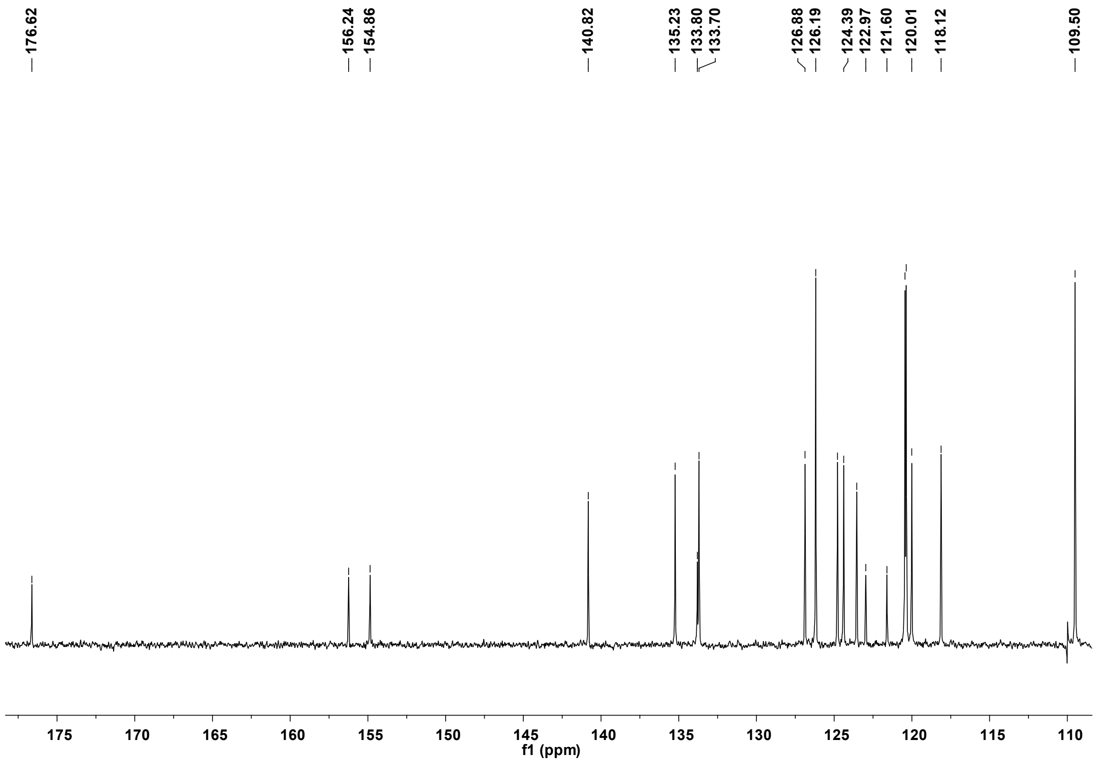

Fig. S4 ^{13}C NMR spectrum of compound CX.

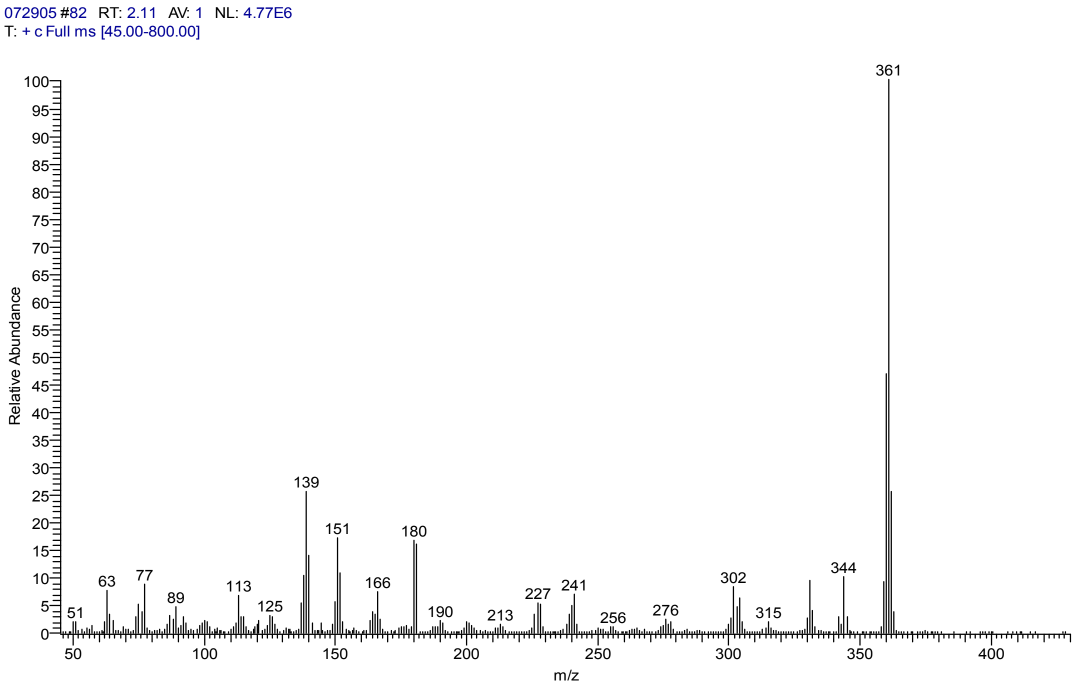

Fig. S5 EI-MS spectrum of compound CX.

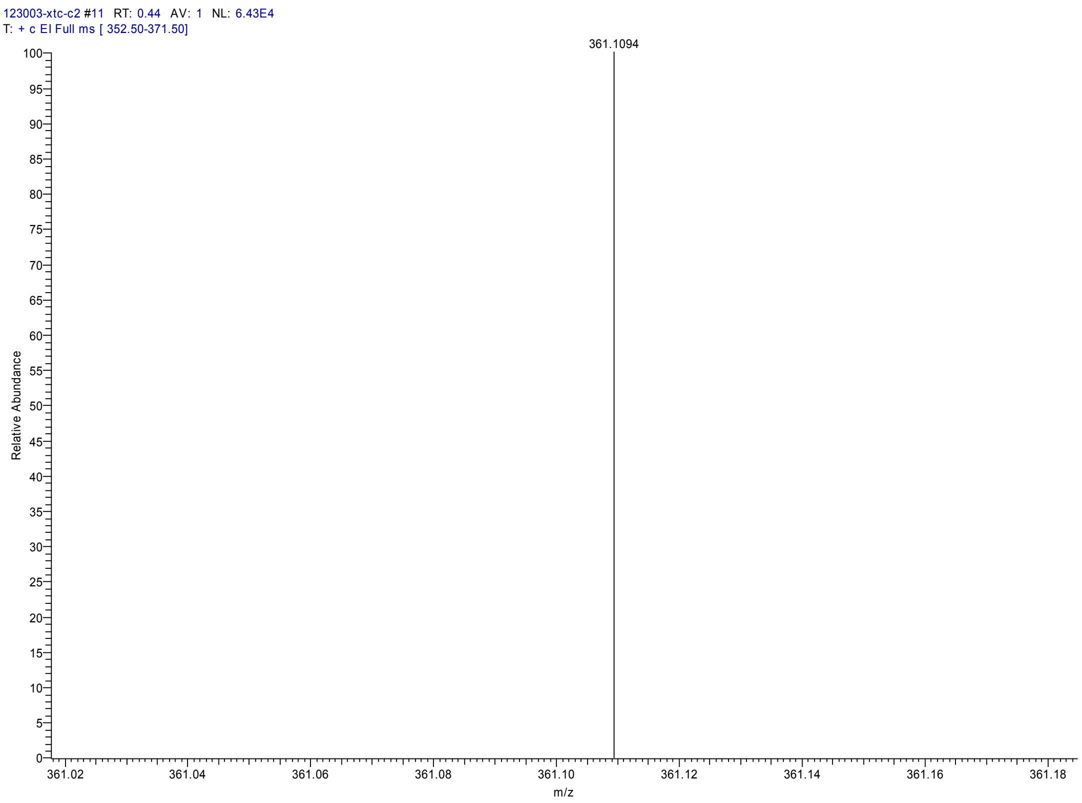

Fig. S6 HRMS spectrum of compound CX.

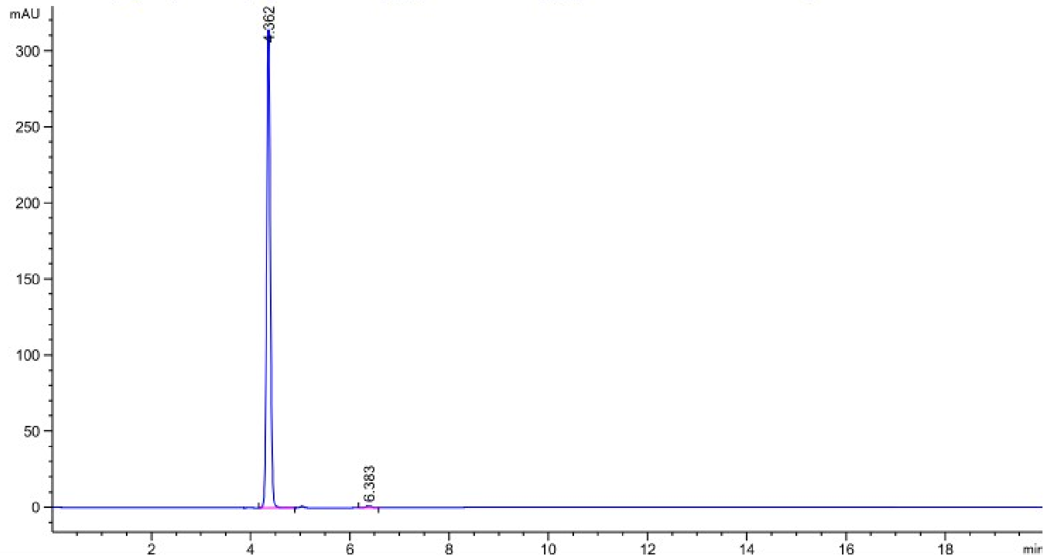

Fig. S7 HPLC measurement for compound CX.

4. Optical Properties

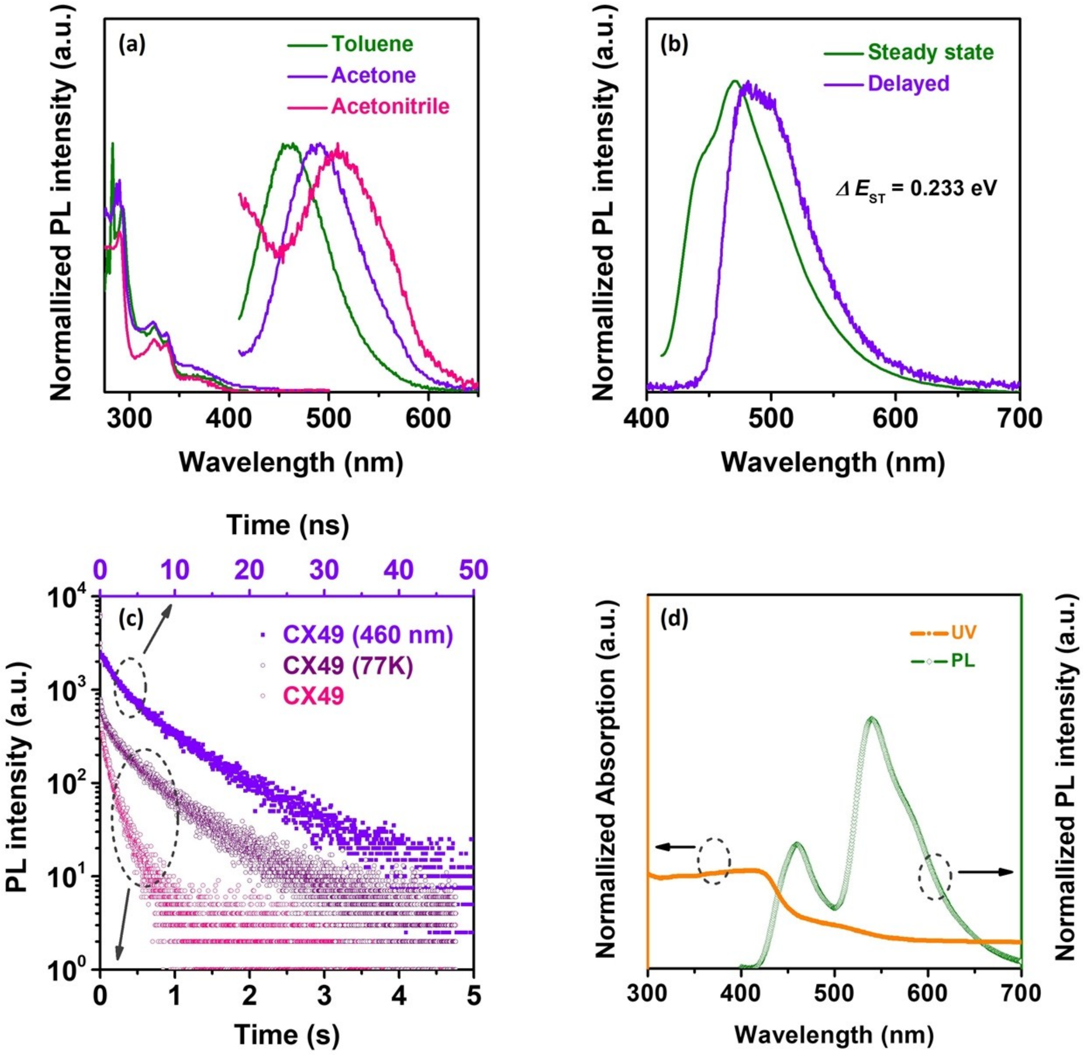

Fig. S8 (a) UV-Vis absorbance and PL spectra of CX in different solvents, (b) Steady-state and delayed (8 ms) PL spectra of CX in toluene solution at 77 K. (c) Time-resolved emission decay curves of CX49 crystal: peak 460 nm (top coordinate), and peak 545 nm (down coordinate). (d) The partial overlap on UV-Vis absorbance and PL spectra of crystal CX49. The concentrations of all solutions were 10^{-5} M and the measurements were performed at room temperature unless otherwise stated.

Table S1 Lifetime (τ) results of CX49 and CX11 crystals at different temperatures.

	CX49		CX11	
Temperature	τ (s)	Intensity (Counts.)	τ (s)	Intensity (Counts.)
77 K	0.57	~5000	0.20	~5000
300 K	0.15	~200	N/A	0

N/A, not application.

Table S2 Lifetime (τ) results of CX49 crystals at different temperatures.

Temperature	Wavelength (nm)	τ_1	τ_2
300 K	460	2.6 ns (35%)	9.7 ns (65%)
300 K	545	0.15 s (100%)	N/A
77 K	485	0.65 s (84%)	0.11 s (16%)

N/A, not application. The data In brackets are the ratio of the lifetime components.

Table S3 Lifetime (τ) results of CX different samples at different temperatures.

Temperature	Wavelength (nm)	τ_1	τ_2
CX49 crystal 300 K	545	0.15 s (100%)	N/A
CX11 crystal 77 K	545	0.07 s (67%)	0.47 s (33%)
Solution 77 K	485	0.30 s (100%)	N/A

N/A, not application. The data In brackets are the ratio of the lifetime components.

5. Time-Dependent Density Functional Theory (TD-DFT) Calculations

The Gaussian 09[1] program was utilized to perform the TD-DFT calculations similar with the previous literature.[2] In order to maintain the specific molecular configurations and corresponding intermolecular locations, the ground state (S_0) geometries of the CX crystal were obtained from the CX11 single crystal structure and no further geometry optimization was conducted. The ground state (S_0) geometries of free CX molecule in vacuum was obtained by geometry optimization based on B3LYP[3]/6-31G (d). The exciton energies of the n-th singlet (S_n) and n-th triplet states (T_n) were obtained on the corresponding ground state geometries using the combination of TD[4]-B3LYP/6-31G (d). Kohn-Sham frontier orbital analysis was obtained for elucidating the mechanisms of possible singlet-triplet intersystem crossings (ISC). The possible S_1 to T_n ISC channels are believed to share part of the same transition orbital compositions, and the energy levels of T_n are considered to lie within the range of $E_{S1} \pm 0.3$ eV. Especially, the major ISC channels are mainly determined based on two elements: (1) the ratio of the same transition configuration in S_1 and T_n states should be large in all the transition orbital compositions, and (2) the energy gap between S_1 and the specific T_n state should be small. When the energy of T_n is lower than S_1, the first element is considered to be more important. The determination of minor ISC channels is vice versa.

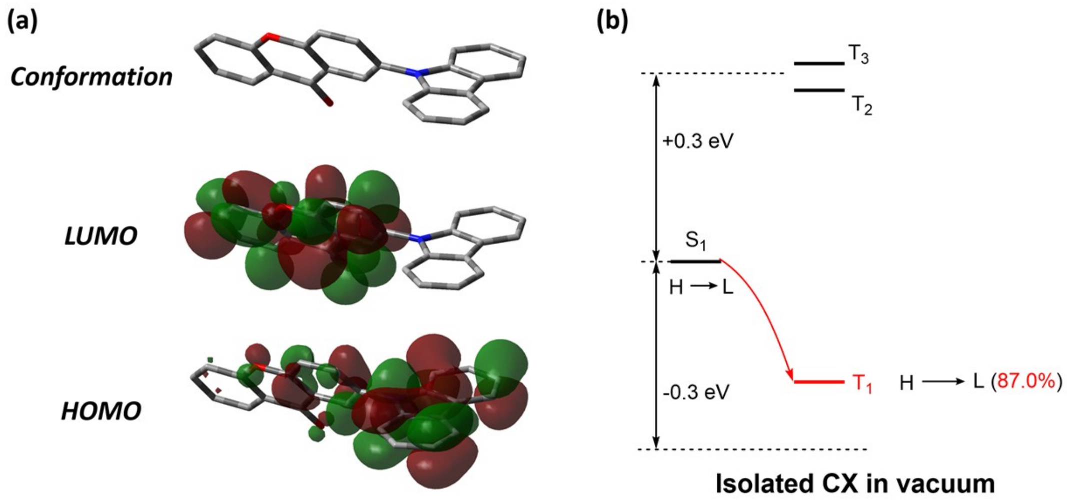

Fig. S9 (a) The conformation and the Kohn-Sham frontier orbitals that are involved in the probable ISC channel obtained from the isolated CX molecule in vacuum. (b) Schematic representations of the TD-DFT calculated energy levels, main orbital configurations and possible ISC channels of isolated CX in vacuum at the singlet (S_1) and triplet (T_n) states. The notations H and L refer to HOMO and LUMO, respectively. The plain arrows refer to the major ISC channel.

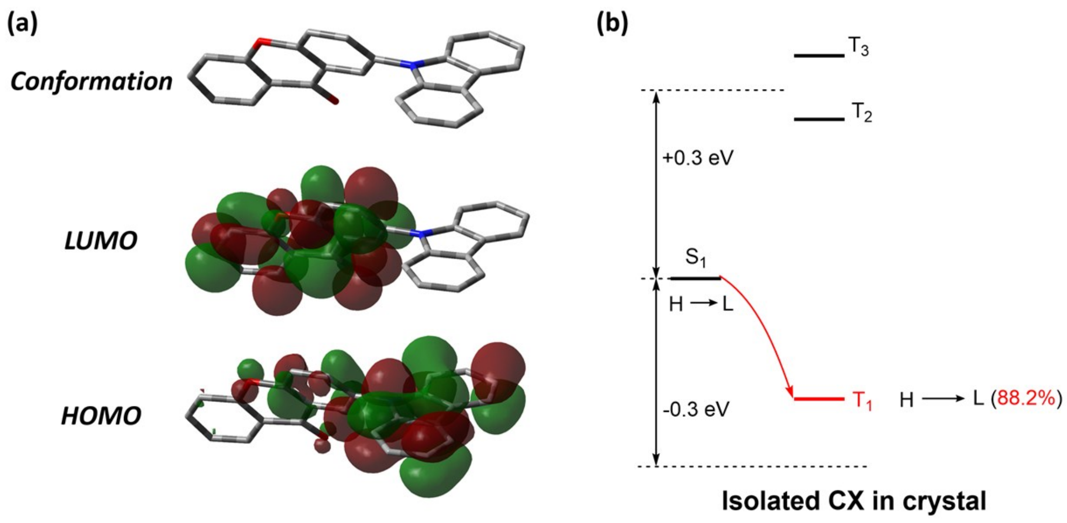

Fig. S10 (a) The conformation and the Kohn-Sham frontier orbitals that are involved in the probable ISC channel obtained from the isolated CX molecule in the CX11 crystal. (b) Schematic representations of the TD-DFT calculated energy levels, main orbital configurations and possible ISC channels of isolated CX in CX11 single crystal at the singlet (S_1) and triplet (T_n) states. The notations H and L refer to the HOMO and

LUMO, respectively. The plain arrows refer to the major ISC channel.

6. Molecular Stacking and Intermolecular Interactions in the Crystalline State

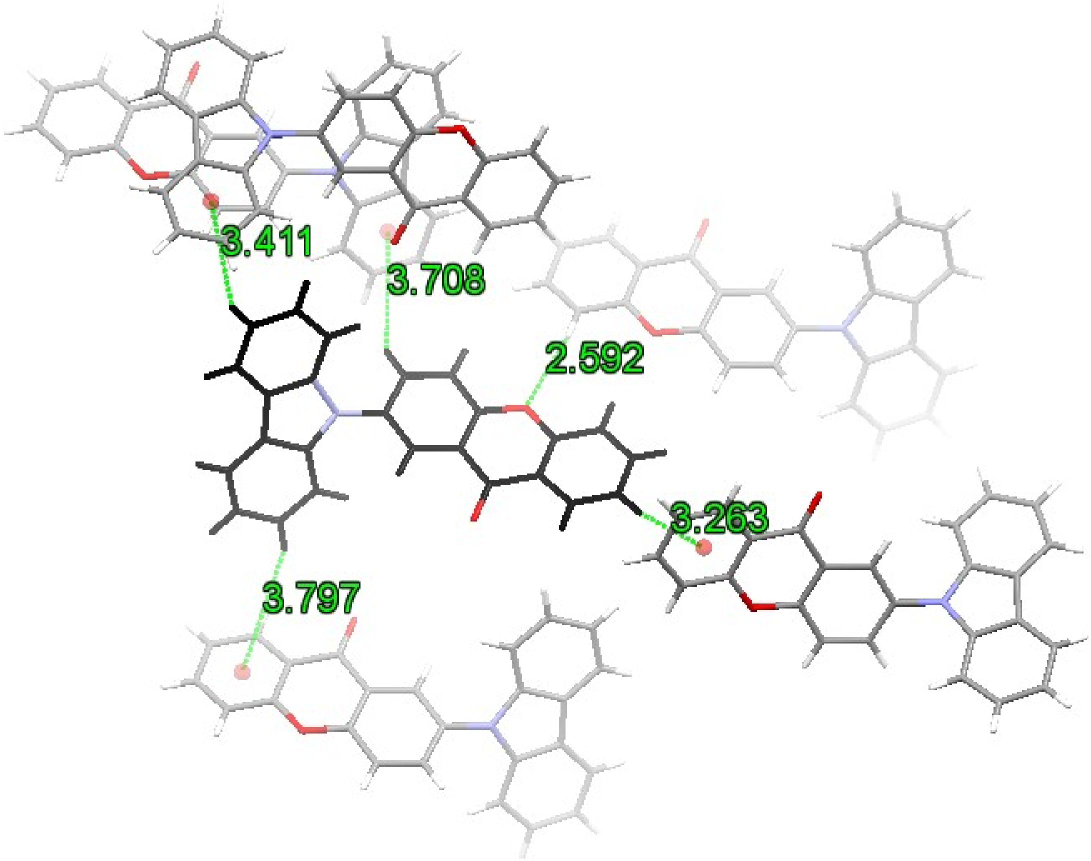

Fig. S11 Intermolecular interactions in the crystal CX11: C-H···π from C-H to the center of near π rings (around 3.263 to 3.797 Å), and H-bond (2.592 Å) between O (xanthone group) and H atoms (another xanthone group of nearby CX molecule).

7. Mechanoluminescence Photos

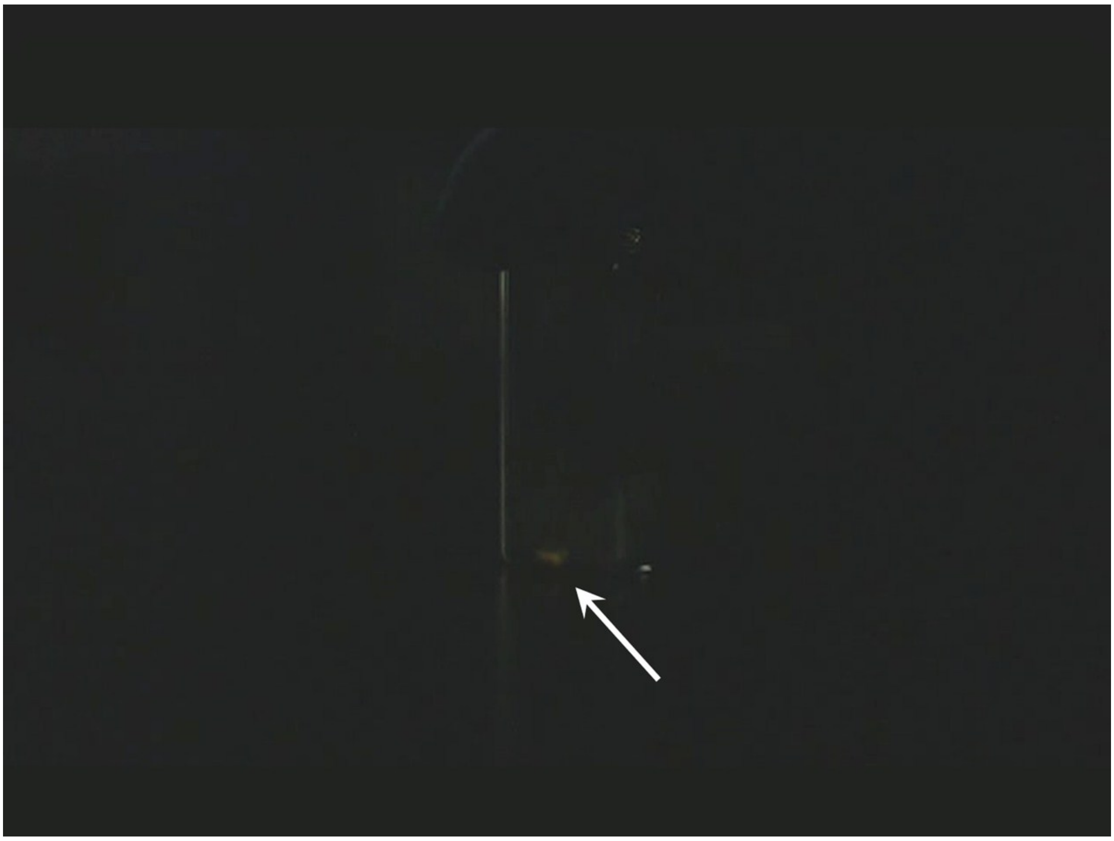

Fig. S12 Photo of mechano-induced phosphorescence of CX49 at room temperature. The sample was simply scratched by a scraper.

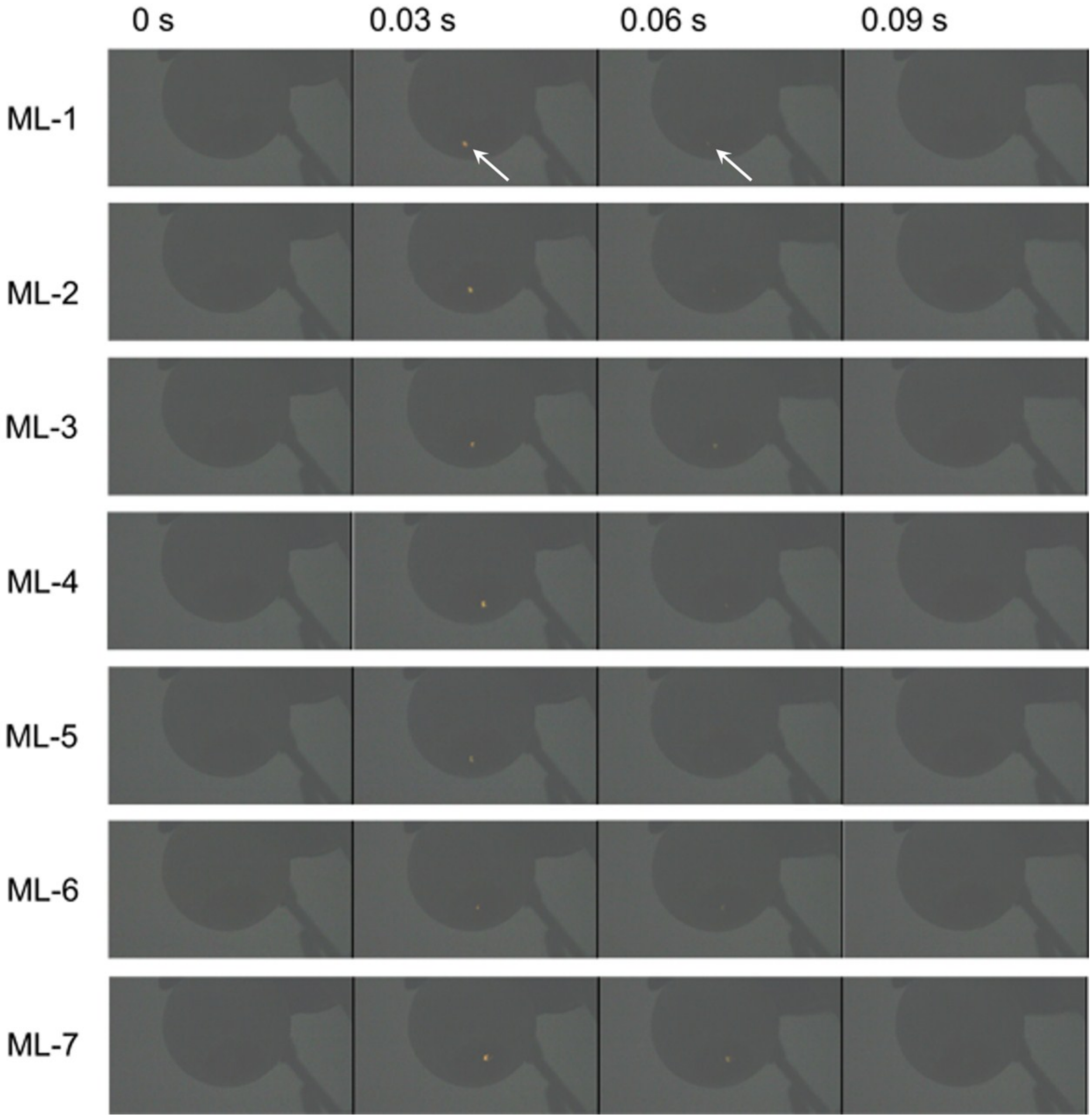

Fig. S13 Photos of mechano-induced phosphorescence of CX49 at 77 K. A light spot can be seen after the scratch stopping (delayed 30 ms) every time. The sample was cooled down to 77K and simply scratched by a scraper. ML-1 means that the sample was scratched for the first time, and so on.

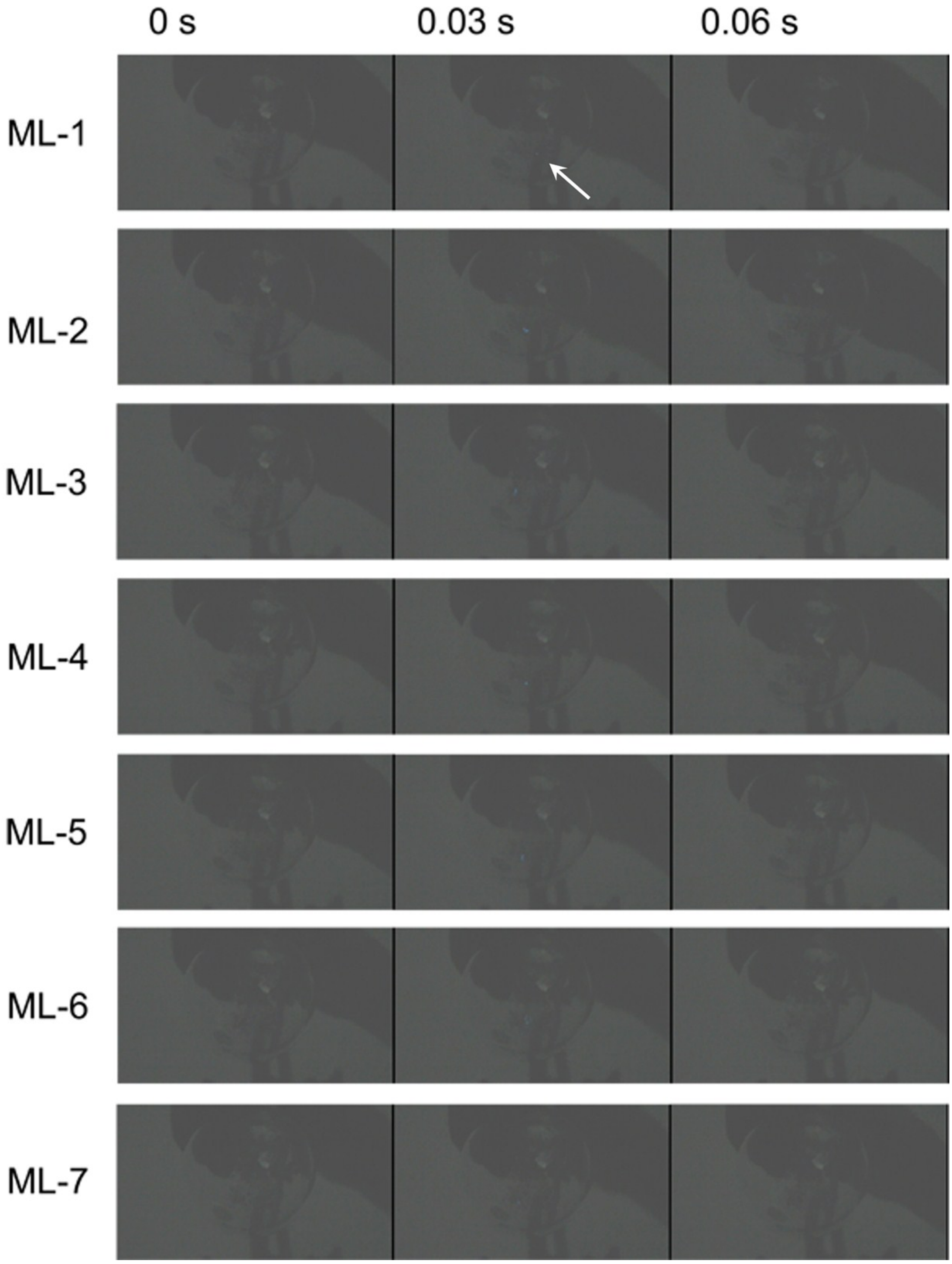
0 s
0.03 s
0.06 s
ML-1
ML-2
ML-3
ML-4
ML-5
ML-6
ML-7

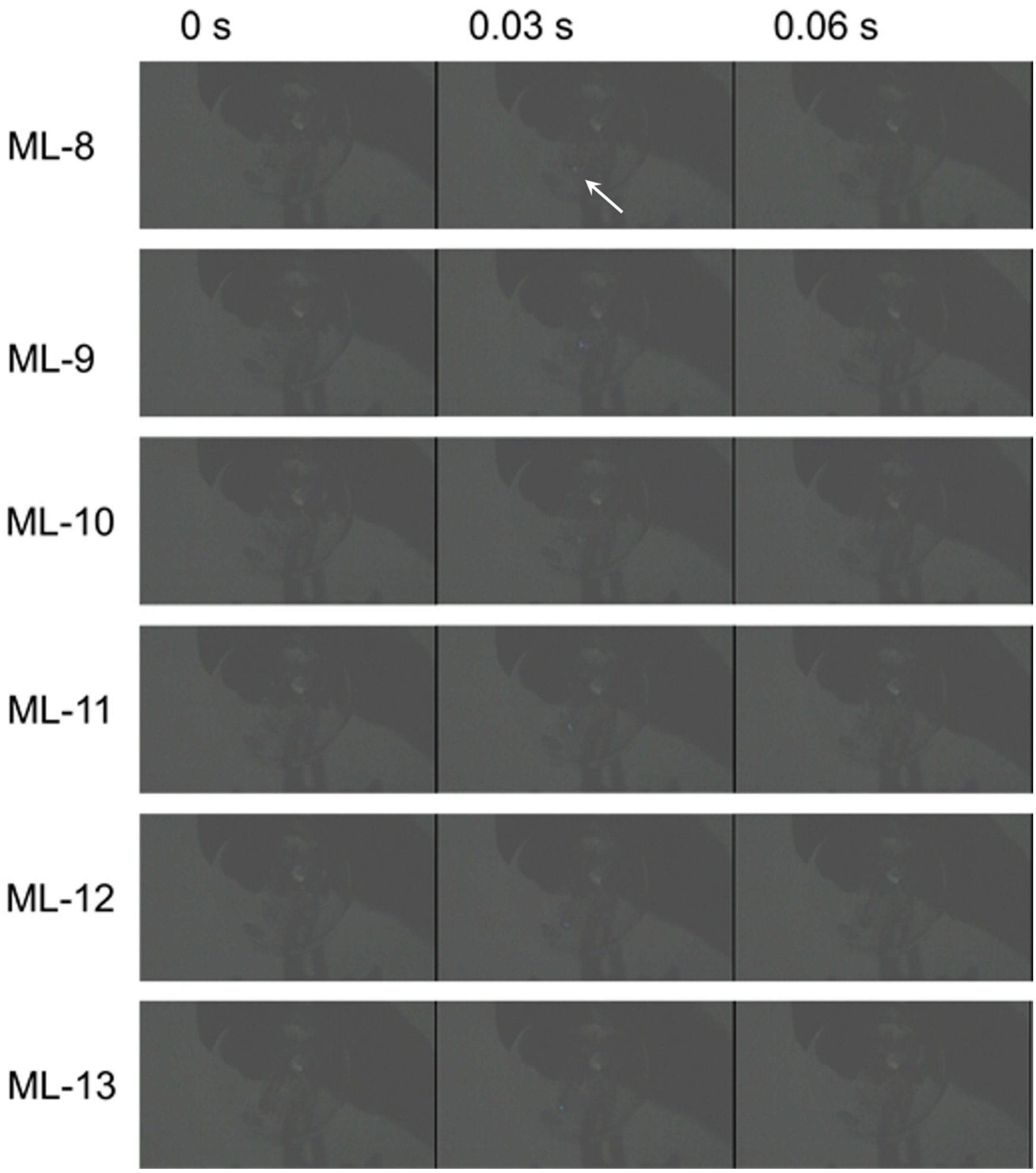

Fig. S14 Photo of mechano-fluorescence of an organic fluorescent material (p-P_4A) from reference at room temperature.[5] No any light spot can be seen after the scratch stopping. The sample was simply scratched by a scraper. ML-1 means that the sample was scratched for the first time, and so on.

8. References

1 M. J. Frisch, G. W. Trucks, H. B. Schlegel, G. E. Scuseria, M. A. Robb, J. R.; Scalmani, G. Cheeseman, V. Barone, B. Mennucci, G. A. Petersson, H. Nakatsuji, M. Caricato, X. Li, H. P. Hratchian, A. F. Izmaylov, J. Bloino, G. Zheng, J. L.; Hada, M. Sonnenberg, M. Ehara, K. Toyota, R. Fukuda, J. Hasegawa, M. Ishida, T. Nakajima, Y. Honda, O. Kitao, H. Nakai, T. Vreven, J. A. Jr. Montgomery, J. E. Peralta, F. Ogliaro, M. Bearpark, J. J. Heyd, E. Brothers, K. N. Kudin, V. N. Staroverov, R. Kobayashi, J. Normand, K. Raghavachari, A. Rendell, J. C. Burant, S. S. Iyengar, J. Tomasi, M. Cossi, N. Rega, J. M. Millam, M. Klene, J. E. Knox, J. B. Cross, V. Bakken, C. Adamo, J. Jaramillo, R. Gomperts, R. E. Stratmann, O. Yazyev, A. J. Austin, R. Cammi, C. Pomelli, J. W. Ochterski, R. L. Martin, K. Morokuma, V. G. Zakrzewski, G. A. Voth, P. Salvador, J. J. Dannenberg, S. Dapprich, A. D. Daniels, Ö. Farkas, J. B. Foresman, J. V. Ortiz, J. Cioslowski and D. J. Fox, *Gaussian 09, Revision A.01*, Gaussian, Inc., Wallingford CT, 2009.

2 Z. Yang, Z. Ma, X. Zhang, D. Ou, Y. Mu, Y. Zhang, C. Zhao, S. Liu, Z. Chi, J. Xu, Y.-C. Wu, P.-Y. Lu, A. Lien and M. R. Bryce, *Angew. Chem. Int. Ed.,* 2016**, 55**, 2181.

3 (a) A. D. Becke, *J. Chem. Phys.,* 1993, **98**, 1372; (b) C. Y. Lee, W. Yang and R. G. Parr, *Phys. Rev. B,* 1988, **37**, 785.

4 R. Bauernschmitt and R. Ahlrichs, *Chem. Phys. Lett.,* 1996, **256**, 454.

5 B. Xu, W. Li, J. He, S. Wu, Q. Zhu, Z. Yang, Y.-C. Wu, Y. Zhang, C. Jin, P.-Y. Lu, Z. Chi, S. Liu, J. Xu and M. R. Bryce, *Chem. Sci.,* 2016, **7**, 5307.
